# Clinical Pharmacist′s Role in Preventing and Managing Drug‐Induced Oral Ulceration: A Bisphosphonate Case in an Older Adult Care Setting

**DOI:** 10.1155/crid/8871258

**Published:** 2025-12-25

**Authors:** Motohiko Sano, Yosuke Iijima, Miki Yamada, Shunsuke Hino, Maho Shinogi, Norio Horie, Takahiro Kaneko

**Affiliations:** ^1^ Laboratory of Clinical Pharmacy Assessment, Hoshi University, Tokyo, Japan, hoshi.ac.jp; ^2^ Department of Oral and Maxillofacial Surgery, Saitama Medical Center, Saitama Medical University, Saitama, Japan, saitama-med.ac.jp

**Keywords:** adverse event, bisphosphonate, older adult care, oral ulcer, pharmacist intervention

## Abstract

Bisphosphonate‐induced oral mucosal ulcers are rare adverse events distinct from medication‐related osteonecrosis of the jaw. This case demonstrates successful conservative management through the modification of drug administration in an older adult care facility. A 91‐year‐old woman with dementia developed bilateral floor of the mouth and sublingual ulceration that persisted for 2 weeks. Histopathological examination excluded malignancy and confirmed a drug‐induced etiology. Management involved correcting the minodronic acid administration technique without discontinuing therapy, which resulted in complete resolution within 4 weeks. This case underscores the importance of appropriate medication administration education and pharmacist involvement in the care of older adults to prevent and manage drug‐related oral diseases.

## 1. Introduction

Bisphosphonates are antiresorptive agents widely prescribed for osteoporosis to prevent skeletal‐related events in patients with cancer [1]. Although medication‐related osteonecrosis of the jaw (MRONJ) is the most recognized adverse oral effect, bisphosphonate‐induced oral mucosal ulcers are a rare but distinct complication [2]. Unlike MRONJ, which involves bone tissue necrosis, these lesions result from direct chemical irritation when tablets remain in the oral cavity, a mechanism similar to drug‐induced esophagitis [3]. In patients with swallowing difficulties, undissolved bisphosphonate tablets may adhere to the mucosa, and dissolution before adequate dilution by saliva causes local chemical injury.

Systematic reviews have indicated that oral bisphosphonate‐induced mucosal disorders are extremely uncommon and typically present as severe ulcerations. They are most often reported in older women, largely associated with inappropriate administration practices, and generally managed through drug discontinuation [4]. Case reports have described mucosal injury caused by direct tablet contact, which is often linked to cognitive impairment or swallowing dysfunction in older adults [5]. Although esophageal ulceration associated with bisphosphonates is well documented, oral mucosal involvement remains poorly recognized among healthcare professionals [6].

The present case is noteworthy because it demonstrates successful conservative management through modifying the administration technique rather than discontinuing therapy. This further underscores the essential role of pharmacists in older adult care facilities where multiple risk factors for drug‐related oral complications converge.

## 2. Case Presentation

A 91‐year‐old Japanese woman residing in an older adult care facility presented with persistent oral mucositis lasting 2 weeks. Her medical history included hypertension, cerebrovascular accident, osteoporosis, and moderate dementia (sufficient for following simple instructions). She had been edentulous for 10 years and required a pure diet because of mild dysphagia. Although her medical history included a cerebrovascular accident, no formal screening for dysphagia was performed. Based on routine observations of meal intake, neither the attending physician nor the caregivers identified significant swallowing concerns beyond the need for texture modification.

Her regular medications included amlodipine 5 mg daily, clopidogrel 75 mg daily, fenofibrate 160 mg daily, minodronic acid 50 mg monthly (initiated 14 months earlier), and eldecalcitol 0.75 *μ*g daily. She had resided in the facility for 3 years and required assistance with activities of daily living.

**Figure 1:**
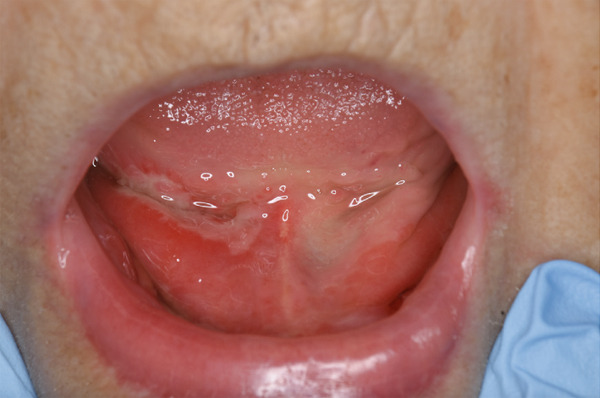
Initial intraoral photograph demonstrating bilateral swelling and erythema with ulceration extending from the anterior floor of the mouth to the sublingual surface.

The patient underwent regular medical examinations performed by a designated attending physician. Prior to referral, the internist conducted an initial evaluation to exclude systemic conditions, such as autoimmune disorders and viral infections. The patient was subsequently referred for oral and maxillofacial surgery for further evaluation of an oral lesion of uncertain etiology. Clinical examination revealed bilateral swelling and erythema extending from the anterior floor of the mouth to the ventral surface of the tongue with scattered superficial ulcerations (Figure 1). The patient experienced pain only upon contact. No exposed bone or sequestrum formation, which is a characteristic of MRONJ, was observed. No signs of salivary gland disease, such as submandibular swelling or abnormalities, were noted in other oral sites or regional lymph nodes. Biopsy was performed to establish a differential diagnosis. The provisional diagnosis at initial consultation was mucositis of uncertain etiology.

Histopathological evaluation of the biopsy tissue revealed an ulcerated squamous epithelium with chronic inflammatory infiltrates, without malignancy, dysplasia, or viral cytopathic effects. *Candida* culture results were negative.

Differential diagnoses included MRONJ (excluded owing to the absence of bone exposure), oral squamous cell carcinoma (excluded by histopathology), autoimmune bullous disorders, viral stomatitis, and traumatic ulceration. The ulceration of the tongue and floor of the mouth improved 2 weeks later, although the erythema persisted. At this stage, the distribution pattern of the lesion (bilateral floor of the mouth and sublingual regions) and the patient′s history of minodronic acid use suggested drug‐induced mucositis, most likely due to intraoral pooling of the tablet in the context of swallowing difficulty. Although caregivers confirmed that the patient remained upright for 30 min after dosing, whether she had consistently swallowed the tablet completely was uncertain.

As the mucosal symptoms improved, bisphosphonate therapy was not discontinued. Caregivers were instructed to ensure complete swallowing of the tablet with adequate water intake (≥ 180 mL), maintain upright positioning, and examine for any residual tablet material in the oral cavity. Supportive care includes gentle oral hygiene and frequent rinsing.

**Figure 2:**
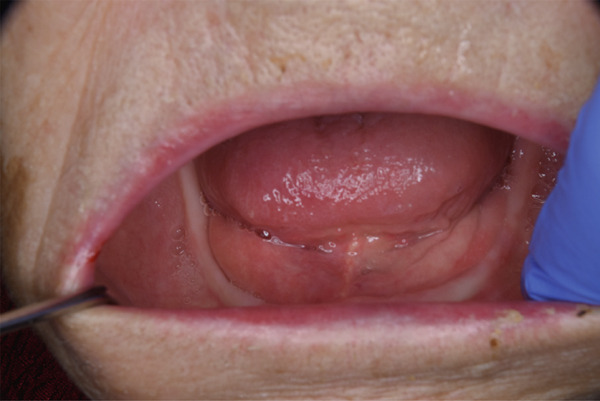
Intraoral photograph after 4 weeks showing complete resolution of oral mucositis and restoration of normal mucosal appearance.

After 4 weeks, the mucositis had resolved completely (Figure 2). The final diagnosis was bisphosphonate‐induced oral mucosal ulceration secondary to intraoral stagnation of minodronic acid. At 4 months of follow‐up, no recurrence or abnormal mucosal findings were observed. Throughout the observation period, no changes in the patients′ concomitant medications (amlodipine, clopidogrel, fenofibrate, and eldecalcitol) occurred, all of which were continued at the same doses.

## 3. Discussion

This case demonstrates that bisphosphonate‐induced oral mucosal ulceration could be managed conservatively without discontinuation of essential osteoporosis therapy. The diagnosis was supported by the temporal association with minodronic acid administration, lesion distribution consistent with intraoral drug stagnation, and marked improvement following the modification of the administration technique. The histopathological and culture results excluded autoimmune bullous diseases, viral infections, and other alternative diagnoses, thereby strengthening the causal link.

Pathophysiologically, the lesion arose from direct chemical irritation when the bisphosphonate tablet was dissolved in contact with the mucosa, a mechanism analogous to that of pill esophagitis and esophageal ulceration [7]. This contrasts with MRONJ, which results from impaired bone remodeling and delayed healing [8]. Tablet size influences acceptability in older adults with swallowing disorders, with tablets < 6.5 mm generally well tolerated and larger tablets poorly accepted [9]. The minodronic acid tablet used in this case, with a major axis of approximately 13 mm, likely exceeded this threshold, thereby increasing the risk of oral stagnation and mucosal injury. Thus, both the intrinsic irritant properties of bisphosphonates and tablet dimensions may contribute to ulcer formation.

Patient‐specific risk factors included advanced age, dementia, and dysphagia, whereas medication‐specific risk factors involved tablet formulation and dosing frequency [9, 10]. The bilateral floor of the mouth and sublingual involvement observed in this study are consistent with gravitational pooling in patients with impaired swallowing. Additionally, although not performed in this case, a formal assessment of the swallowing function may be warranted in similar patients. The Repetitive Saliva Swallowing Test is a simple, noninvasive screening tool that can be easily implemented in older adult care facilities for the general evaluation of swallowing function [11].

Published cases report drug discontinuation as the predominant management strategy, accounting for approximately 85.7% of the cases [4]. However, this case illustrates that modification of the administration technique alone may be sufficient. Discontinuation was carefully considered; however, severe osteoporosis and elevated fracture risk necessitated continued treatment. The localized nature of the lesion and absence of systemic toxicity further supported a conservative approach centered on administration modification rather than withdrawal of therapy.

Pharmacist involvement is particularly important in older adult care facilities where cognitive impairment and polypharmacy increase the risk of medication‐related adverse events [12]. A wide range of drugs, including chemotherapeutic agents, methotrexate, nonsteroidal anti‐inflammatory drugs, nicorandil, angiotensin‐converting enzyme inhibitors, and antidepressants, can cause oral ulceration [13]. Moreover, many medications exacerbate dysphagia and xerostomia, which promotes oral drug stagnation [14]. In Japanese care facilities, pharmacists collaborate with the nursing staff to manage complex drug regimens and play a key role in recognizing and preventing oral complications [15]. This case highlights the importance of pharmacists′ expertise in differential diagnosis, treatment planning, and caregiver education. Specifically, when prescribing bisphosphonates, clinicians should assess swallowing function, cognitive status, and oral mucosal conditions to identify high‐risk patients. Appropriate administration instructions should be provided to patients and caregivers, and medication adherence and oral cavity status should be regularly monitored. For patients with swallowing difficulties, pharmacists should propose alternative formulations, such as liquid preparations or injectable agents, to prescribing physicians. In these interventions, pharmacists can leverage their expertise in drug characteristics including tablet size, mucosal irritancy, and the availability of alternative formulations and serve as key links among physicians, dentists, and caregiving staff.

The clinical implications of this case include maintaining a high index of suspicion for drug‐induced mucosal lesions in patients receiving bisphosphonates, especially in those with cognitive impairment or swallowing difficulties. Early recognition and conservative management can preserve the systemic therapeutic benefits while ensuring local mucosal recovery. Although no standardized long‐term monitoring protocol currently exists for bisphosphonate‐induced oral mucosal ulceration, facilities may consider implementing a “high‐risk watch list” for patients receiving bisphosphonates who have swallowing difficulties or cognitive impairment. This system ensures heightened vigilance during medication administration and periodic oral cavity inspection, thereby potentially preventing recurrence.

This study is limited by its single‐case design restricting generalizability, incomplete exclusion of potential confounders, and a short follow‐up period of 4 months. Nevertheless, a thorough diagnostic evaluation, clearly documented timeline, and successful conservative management provide meaningful clinical insights. Future initiatives should include the development of standardized written protocols and educational materials for bisphosphonate administration in older adult care settings that could further enhance caregiver competence and patient safety.

This case demonstrates that even when caregivers follow standard positioning instructions, incomplete swallowing could result in adverse outcomes. Comprehensive education covering all aspects of safe administration may enhance caregivers′ confidence in patient safety.

## 4. Conclusions

Bisphosphonate‐induced oral mucosal ulcers could be effectively managed by modifying the administration technique without discontinuing the therapy. Active pharmacist involvement in older adult care facilities as part of multidisciplinary teams is essential for preventing and managing drug‐related oral complications. Clinicians should consider medication‐related etiologies when evaluating oral lesions in older adults receiving bisphosphonates, particularly in those with cognitive or swallowing impairments.

## Consent

The authors certify that they have obtained appropriate patient consent. Upon signing the form, the patient consented to her images and other clinical information being reported in the journal.

## Conflicts of Interest

The authors declare no conflicts of interest.

## Author Contributions

Motohiko Sano conceptualized and supervised the study, wrote the original draft, and revised and edited the manuscript. Yosuke Iijima, Miki Yamada, and Norio Horie conceptualized and supervised the study and revised and edited the manuscript. Shunsuke Hino, Maho Shinogi, and Takahiro Kaneko acquired data and revised and edited the manuscript.

## Funding

No funding was received for this manuscript.

## Data Availability

Data supporting the study findings are available from the corresponding author upon request. The data are not publicly available because of privacy and ethical restrictions.
